# Inconsistency in Diarrhea Measurements when Assessing Intervention Impact in a Non-Blinded Cluster-Randomized Controlled Trial

**DOI:** 10.4269/ajtmh.18-0872

**Published:** 2019-06-03

**Authors:** Nusrat Najnin, Karin Leder, Andrew Forbes, Leanne Unicomb, Firdausi Qadri, Pavani K. Ram, Peter J. Winch, Farzana Begum, Shwapon Biswas, Tahmina Parvin, Farzana Yeasmin, Alejandro Cravioto, Stephen P. Luby

**Affiliations:** 1 International Centre for Diarrheal Disease Research, Bangladesh (icddr,b), Dhaka, Bangladesh;; 2 Department of Epidemiology and Preventive Medicine, School of Public Health and Preventive Medicine, Monash University, Melbourne, Australia;; 3 University at Buffalo, Buffalo, New York;; 4 Johns Hopkins Bloomberg School of Public Health, Baltimore, Maryland;; 5 Department of Medicine, Rangpur Medical College Hospital, Rangpur, Bangladesh;; 6 Facultad de Medicina, Universidad Nacional Autónoma de México, Ciudad de México, México;; 7 Stanford University, Stanford, California

## Abstract

To explore the consistency in impact evaluation based on reported diarrhea, we compared diarrhea data collected through two different surveys and with observed diarrhea-associated hospitalization for children aged ≤ 5 years from a non-blinded cluster-randomized trial conducted over 2 years in urban Dhaka. We have previously reported that the interventions did not reduce diarrhea-associated hospitalization for children aged ≤ 5 years in this trial. We randomly allocated 90 geographic clusters comprising > 60,000 low-income households into three groups: cholera vaccine only, vaccine plus behavior change (cholera vaccine and handwashing plus drinking water chlorination promotion), and control. We calculated reported diarrhea prevalence within the last 2 days using data collected from two different survey methods. The “census” data were collected from each household every 6 months for updating household demographic information. The “monthly survey” data were collected every month from a subset of randomly selected study households for monitoring the uptake of behavior change interventions. We used binomial regression with a logarithmic link accounting for clustering to compare diarrhea prevalence across intervention and control groups separately for both census and monthly survey data. No intervention impact was detected in the census (vaccine only versus control: 2.32% versus 2.53%; *P* = 0.49; vaccine plus behavior change versus control: 2.44% versus 2.53%; *P* = 0.78) or in the vaccine only versus control in the monthly survey (3.39% versus 3.80%; *P* = 0.69). However, diarrhea prevalence was lower in the vaccine-plus-behavior-change group than control in the monthly survey (2.08% versus 3.80%; *P* = 0.02). Although the reasons for different observed treatment effects in the census and monthly survey data in this study are unclear, these findings emphasize the importance of assessing objective outcomes along with reported outcomes from non-blinded trials.

## INTRODUCTION

Diarrhea is among the top five leading causes of total years of life lost globally.^1^ It is still a major cause of child mortality and morbidity in low-income countries.^2–5^ Most of the pathogens that cause diarrhea are transmitted via the fecal–oral route.^6,7^ Interventions that improve the quality of drinking water, sanitation, and hygiene (WASH) behavior can potentially interrupt transmission and reduce diarrhea.^8–11^ One of the commonly used indicators to assess effectiveness of these environmental interventions is reported diarrhea.^10–12^ For example, a systematic review of 45 cluster-randomized controlled trials for assessing effectiveness of improving water quality for diarrhea reduction shows that the primary outcome in all of these studies was reported diarrhea.^12^ Data collectors usually collect this information by regularly visiting study households and asking an adult participant to recall diarrhea episodes experienced by household members within recent days or weeks.^13^ Measuring diarrhea objectively such as by observing diarrhea-associated hospital admissions or by complementing disease reporting with microbiological testing of stool for specific microorganisms is prone to less subjective reporting bias, and hence is a preferred way of measuring diarrhea compared with reported outcomes.^14^ However, these approaches require larger study sizes to capture these less common outcomes and are more complex and costly, and so are deployed less commonly.^15–19^ Concerns raised regarding reliability of reported diarrhea include courtesy bias,^20,21^ imperfect and biased recall,^22–27^ and surveillance fatigue.^28–30^ In addition, there is concern about the reliability of measuring subjective health outcomes in non-blinded trials due to observer bias.^31^ A systematic review of 21 randomized clinical trials with blinded and non-blinded assessment of the same binary outcome showed that the non-blinded assessors of subjective binary outcomes generated substantially biased effect estimates.^32^ Because of these concerns, in some non-blinded trials, a reduction of diarrhea by even 50% may not necessarily be due to a true intervention effect.^33^ To overcome this, it is now recommended that in studies where blinding is not possible, there should be at least one objectively assessed outcome even if the primary outcome is subjective.^14^ Alternatively, validation studies for estimating the degree of bias should be incorporated to improve data interpretation.^34^

In 2011, we conducted a cluster-randomized trial over 2 years among > 60,000 low-income households in urban Dhaka, Bangladesh, to evaluate the impact of oral cholera vaccine along with handwashing and water treatment interventions in reducing diarrhea, including cholera.^35,36^ In this study, non-blinded assessors collected reported diarrhea data using similar construction of questions for children aged ≤ 5 years using two separate surveys, each of which was conducted on the same study population throughout the study period; data on diarrhea-associated hospitalization were also collected for children aged ≤ 5 years. We have previously reported that neither cholera vaccination alone nor cholera vaccination combined with behavior change intervention efforts measurably reduced observed diarrhea-associated hospitalization among children aged ≤ 5 years.^36^ In this current study, we aimed to compare whether data collected using two different survey methodologies, carried out by different data collection teams to elicit reported diarrhea, impacted on the interpretation of intervention effects on measured reported diarrhea among children aged ≤ 5 years. We also compared the reported diarrhea data with objectively measured diarrhea-associated hospitalization rates for children aged ≤ 5 years in the same study. We hypothesized that in this non-blinded trial, the interpretation of impact evaluation based on reported diarrhea data collected through two different surveys for children aged ≤ 5 years will be similar.

## MATERIALS AND METHODS

### Trial design, study setting, and participants.

We analyzed data from a cluster-randomized control trial conducted in densely populated (∼17,000 people living/km^2^) low-income communities of the Mirpur area of urban Dhaka between 2011 and 2013. In these communities, households are commonly organized into compounds (usual number of households in a compound: ∼20–25; range: 2–100), with individual families often renting a small room and several households sharing a common water source, kitchen, and toilet. Details regarding the trial design, participant selection, and interventions have been described previously.^35,36^ Briefly, we applied criteria including low per-capita income, sharing water source, poor sanitation, and poor living conditions to select high-risk, diarrhea-prone study areas, which were then divided into 90 geographic clusters. Each cluster was surrounded by a 30-m buffer zone to limit contamination of the interventions across clusters. A statistician external to the International Centre for Diarrhoeal Disease Research, Bangladesh (icddr,b), randomly assigned the geographic clusters into three study groups: 1) cholera vaccine alone group (denoted as “vaccine-only” group hereafter); 2) combined cholera vaccine and behavior change communication intervention group (denoted as “vaccine-plus-behavior-change” group hereafter); and 3) control group (continued standard habits and practices).

### Study interventions and blinding.

The study interventions were as follows: 1) cholera vaccine: two doses of killed whole-cell, oral cholera vaccine, ShanChol^™^ (Shantha Biotechnics-Sanofi, India), were administered 14 days apart to participants who were non-pregnant and children aged > 1 year; and 2) promotion of handwashing with soap and drinking water chlorination, both implemented at the compound level near the shared water source. Behavior change interventions to improve handwashing and point-of-use water treatment included enabling both hardware and behavior change communication messages. Hand-washing hardware consisted of a 30-L water tank with a tap, a bowl where rinse water could accumulate, and soap/soapy water.^37^ Point-of-use water treatment hardware consisted of a chlorine dispenser containing liquid sodium hypochlorite. The behavior change strategy was developed following the Integrated Behavioral Model for WASH theoretical framework.^38^ Dushtha Shasthya Kendra (DSK), a nongovernmental organization with considerable experience working on WASH issues in Mirpur, delivered the behavioral interventions.

Blinding was not possible in this study because of the nature of the interventions.

### Data collection.

Two different teams of icddr,b employees having similar employment status and educational qualifications worked independently of each other to collect reported diarrhea data concurrently among children aged ≤ 5 years from the same study population over the 2-year study period. Two different surveys were used:

a. Census: A team of approximately 30 data collectors collected census data every 6 months from each house in the study area. The primary aim of census was to collect information on births, deaths, and in- and out-migration of individuals in the study area. During each visit, data collectors also asked respondents about each family member, including children aged ≤ 5 years, to ascertain whether anyone had had “diarrhea within last 48 hours.” Interviewers explained that ≥ 3 loose stools within 24 hours would be considered to constitute diarrhea.

The census data collection team members were recruited and trained by the icddr,b researchers who were responsible for overseeing cholera vaccine–related activities in the field. Most of the data collectors in this team had experience working on vaccine trials. The training continued for 4 weeks for this group. On average, each data collector visited ∼30 households each day, usually requiring ∼15 minutes for completion of data collection from each household.

b. Monthly survey: 400 households were randomly selected each month from the most updated census database. This random selection was carried out at the household level and not at the cluster level. Each month a team of approximately 11 data collectors collected data from a different set of 200 randomly selected study households in the vaccine-plus-behavior-change group, and 100 households in each of the vaccine-only and control groups. The sample size calculation was carried out for the primary aim of the original study and not for this sub-study. The monthly assessment of 400 households was designed to be low enough to be logistically manageable, but to provide representative real-time trend data on intervention uptake. This selection process was predefined in the study protocol.

The main goal of monthly surveys was monitoring of uptake of behavioral interventions. This involved asking questions about hand-washing and drinking water treatment behaviors, observing hand-washing practices among study participants, spot-checking for the presence of soap and water at hand-washing stations and for liquid chlorine in chlorine dispensers, and spot-checking for the presence of residual chlorine in stored drinking water using Hach colorimeter (HACH LANGE GmbH, Germany) if the households reported treating water with chlorine. Data collectors also asked the respondents about each of the family members, including children aged ≤ 5 years, to determine if they had “diarrhea within last 2 days.” Interviewers also explained that ≥ 3 loose stools within 24 hours would be considered to constitute diarrhea. Data collectors were instructed to collect information on diarrhea at the beginning of the interview to reduce bias, as asking about diarrhea and intervention products occurred at the same visit. The study households were typically arranged as compounds, and because data collectors visited randomly selected households from these compounds every month, they visited some of the compounds several times during the 2-year study period. The time interval between the visits in these compounds varied from a few days to a few months.

The monthly data collectors were recruited and trained by icddr,b researchers who were responsible for quantitative assessment of uptake of the behavioral interventions. These researchers were also involved in designing and implementing the behavioral interventions. Most data collectors had previous experience in collecting behavioral intervention–related data. This team received training for 2 weeks before data collection started. In a typical day, they were able to interview ∼4 householders, usually requiring ∼45–90 minutes for completion of each interview.

c. A separate team of data collectors collected information on diarrhea-associated hospitalization for children aged ≤ 5 years from 12 governmental and nongovernmental study hospitals/clinics with inpatient facilities in and around the study area. Details of this study have been reported previously.^35,36^

### Qualitative data collection on the training and field experience of census and monthly survey data collection teams.

We conducted two group discussions among seven census data collectors and six monthly survey data collectors in the local Bengali language. Our aim was to understand the similarities and differences in their training and data collection procedures, focus of data collection, and data collection experiences in the field that could have affected the reported diarrhea data collected by them. The group discussions lasted for ∼45–60 minutes, and data were captured with a digital audio recorder. We also interviewed data collection supervisors from each team separately for cross-checking the information provided by the data collectors.

### Study timeline.

For all data analyses, we considered the study period from October 2011 to July 2013. During this time, both cholera vaccine and behavior change interventions had already been implemented.

### Data analysis of diarrhea reporting.

Because of the case definition that we used in both surveys, diarrheal illness of any severity, including cholera cases, might have been included in the analysis. We calculated and compared reported diarrhea prevalence for children aged ≤ 5 years across intervention and control groups separately for both census and monthly survey data. To compare the overall and intervention group–specific reported diarrhea prevalence in census and in monthly surveys, we used binomial regression with a logarithmic link to calculate differences in prevalence with robust standard errors to account for clustering.

### Data analysis of diarrhea-associated hospitalization.

Details about data analysis related to diarrhea-associated hospitalization for children aged ≤ 5 years have been published elsewhere.^36^ In short, from the census data, we identified people who migrated in or out of the study area during the study period. We calculated the incidence of diarrhea-associated hospitalization for children aged ≤ 5 years during the study period by counting the number of admissions in each group, and by summing the person-time that study participants contributed for each trial group. We adjusted the hospitalization incidence for the cluster-randomized design of the trial using robust “sandwich” variance estimators.

### Qualitative data analysis.

We summarized each interview after transcribing the audio recordings into English. We then manually analyzed the data by compiling under themes, such as training experience for collecting data, focus of data collection, field experience in collecting data including frequency of visits in compounds, and involvement of data collectors with study participants in dealing with problems related to behavior change intervention materials. We then examined the similarities, differences, and connections between each theme.

### Ethical consideration.

An adult study participant from each household provided informed written consent. Confidentiality was maintained by keeping data anonymous throughout the study period and during analysis. The Institutional Review Board of the International Vaccine Institute, and the Research Review Committee and the Ethical Review Committee of icddr,b, Dhaka, Bangladesh, reviewed and approved the study protocol. The study was registered at ClinicalTrials.gov (Registration number: NCT01339845).

## RESULTS

Data from 22 monthly surveys and four census surveys were analyzed.

Demographic and household characteristics of enrolled study participants were similar across the groups, except for the presence of sanitary latrines (latrine with piped sewer system/septic tank, pit latrine with slab plus water seal, pit latrine with slab and no water seal but with lid, ventilated improved pit latrine, dual pit latrine, or composting toilet), which was slightly lower in the vaccine-only group (Table 1). The age-stratified distribution of study participants was similar across the groups in both census and monthly surveys (Supplemental Tables 2 and 3).

**Table 1:** Characteristics of individuals and households across the intervention groups during the study period (October 2011–July 2013)*

Characteristics of individuals	Vaccine-only group (*n* = 142,879) (%)	Vaccine-plus-behavior-change group (*n* = 140,202) (%)	Control group (*n* = 137,451) (%)
Age, mean (SD) (years)	22.8 (15.4)	22.8 (15.3)	22.8 (15.5)
≤ 5	14.7	14.7	14.8
> 5–15	18.3	17.8	18.5
> 15–50	62.1	62.6	61.5
> 50	4.9	4.9	5.2
Gender (male)	48.3	48.7	48.6
Educational status			
No formal education (includes children aged < 5 years)	44.3	40.7	42.9
Below primary	17.0	17.0	17.0
Primary and some secondary	30.6	32.4	30.9
Above secondary	8.1	9.9	9.3
No. of people in a family (median, interquartile range)	5 (4–6)	5 (3–6)	5 (4–6)
No. of months living in this house (median, interquartile range)	5 (2–36)	6 (2–36)	6 (3–36)

Characteristics of households	Vaccine group (*n* = 42,217) (%)	Vaccine-plus-behavior-change group (*n* = 42,215) (%)	Control group (*n* = 39,738) (%)
Source of drinking water (municipal piped water supply)†	99.9	99.8	99.9
Treat drinking water (yes)	53.2	64.2	56.2
Boil water	52.0	58.7	54.8
Filter water	0.8	1.2	0.9
Chemical treatment	0.4	4.3	0.4
Shared kitchen (yes)	91.6	95.0	91.2
Shared toilet (yes)	97.2	96.6	96.6
Type of toilet (direct observation)			
Sanitary latrine with or without flush‡	72.8	85.3	84.0
Non-sanitary	27.2	14.8	16.0
Waste disposal (fixed place)	84.1	88.3	83.4
House construction material			
Roof			
Corrugated iron	85.0	81.7	79.7
Brick/concrete	14.8	18.7	20.2
Bamboo/wood/other	0.2	0.1	0.1
Floor			
Brick/concrete	92.0	92.7	92.9
Bamboo/wood/other	8.0	7.3	7.1
Wall			
Corrugated iron	28.6	21.9	24.8
Brick/concrete	69.2	76.6	72.5
Bamboo/wood/other	2.3	1.4	2.6
No. of rooms in the house, mean (SD)	1.1 (0.4)	1.1 (0.4)	1.1 (0.4)

* Unique person/household identification number; some categories do not sum to 100% because of rounding.

† Other sources of drinking water include well, bottled water, water vendor, and pond/canal/river.

‡ Latrine with piped sewer system, septic tank, pit latrine with slab plus water seal, pit latrine with slab and no water seal but with lid, ventilated improved pit latrine, dual pit latrine, or composting toilet.

### Reported diarrhea prevalence.

The control group had the highest diarrhea prevalence in both census and monthly surveys during the study period. Diarrhea prevalence was lower in the vaccine-plus-behavior-change group than the control group in the monthly survey (2.08% versus 3.80%; *P* = 0.02) but not in census data (2.44% versus 2.53%; *P* = 0.78) (Table 2). Diarrhea prevalence was slightly lower in the vaccine-only group than in the control group in both census and monthly surveys, but the difference was not statistically significant (Table 2).

**Table 2:** Reported diarrhea prevalence within last 2 days of interview among children aged ≤ 5 years across intervention and control groups from census and monthly survey data (October 2011–July 2013)

Groups	Diarrhea prevalence from census, % (*n*/*N*) (95% CI)	Intervention vs. control groups in census,* % of difference in prevalence; 95% CI; *P*-value	Diarrhea prevalence from monthly surveys, % (*n*/*N*) (95% CI)	Intervention vs. control groups in monthly surveys,* % of difference in prevalence; 95% CI; *P*-value
All study groups combined	2.43 (6,081/250,514) (2.19, 2.69)	–	2.87 (171/5,949) (2.26, 3.65)	–
Vaccine-only group	2.32 (1,981/85,484) (1.91, 2.81)	Prevalence 0.2% lower in vaccine-only group than control; −0.0080, 0.0039; 0.49	3.39 (53/1,564) (2.06, 5.53)	Prevalence is 0.4% lower in vaccine-only group than control; −0.0244, 0.0162; 0.69
Vaccine-plus-behavior-change group	2.44 (2,028/83,075) (2.03, 2.94)	Prevalence 0.1% lower in vaccine-plus-behavior-change group compared to control; −0.0068, 0.0051; 0.78	2.08 (59/2,832) (1.39, 3.12)	Prevalence is 1.7% lower in vaccine-only group than control; −0.0320, −0.0023; 0.02
Control group	2.53 (2,072/81,955) (2.12, 3.01)	–	3.80 (59/1,553) (2.67, 5.37)	–

* Results are adjusted for cluster-randomized design.

**Figure 1.**
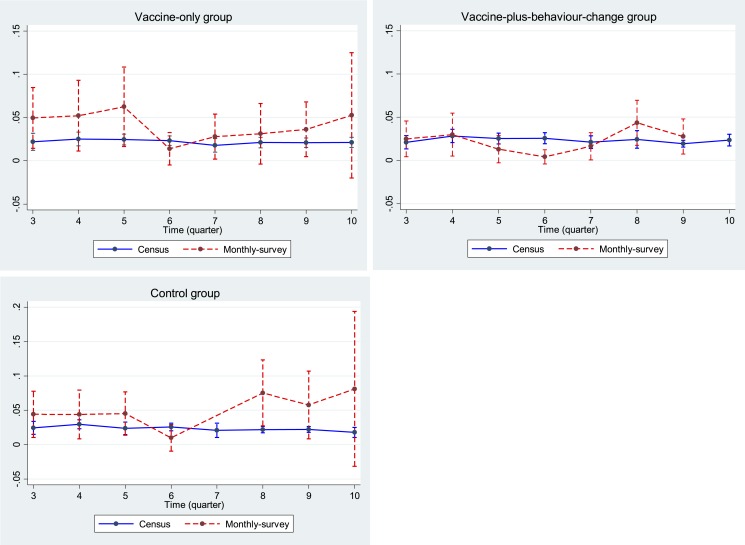
Comparison of reported diarrhea prevalence between census and monthly surveys (along with 95% CI) for children aged ≤ 5 years across intervention and control groups during the study period. This figure appears in color at www.ajtmh.org.

Diarrhea prevalence in each quarter over the 2-year study period was mostly higher in the monthly survey than census in the vaccine-only and control groups, but not in the vaccine-plus-behavior-change group (Figure 1). However, the 95% CIs of the census and monthly survey diarrhea prevalence in each quarter across all groups mostly overlapped each other (Figure 1), indicating that the diarrhea prevalence in the census was not very different from the prevalence in the monthly surveys during the study period.

### Hospitalization rate for children aged ≤ 5 years.

Results on objectively measured diarrhea-associated hospitalization rate for children aged ≤ 5 years have been published elsewhere.^36^ Briefly, we observed no impact of interventions on the diarrhea-associated hospitalization rate (hospitalization rate in groups: vaccine only: 39.3/1,000 person-years; vaccine plus behavior change: 43.3/1,000 person-years; control: 39.4/1,000 person-years) (Supplemental Table 1).

### Qualitative feedback on data collectors’ training and field experience.

In the group discussions, both census and monthly survey data collectors mentioned that the trainers first discussed the research objectives with them and then discussed each of the items from the questionnaires until the data collectors were clear about all aspects. The data collectors then practiced mock interviews with each other and piloted the questionnaires in the field. If they had feedback about any item in the questionnaire, the trainers addressed this by discussing or revising it. Finally, when they were clear and confident about the data collection instrument, they began data collection for the study. Both census and monthly survey data collectors received extensive training about how to identify the correct households in the study area using the geographic information system. In addition, the census team was also trained on identifying and updating household information if there was any in- or out-migration in the study area. If a new data collector joined the team, that person was given similar training by the same trainers, and then he/she was attached with another data collector in the field for several days until the person was confident enough to collect data on his/her own.

Data collectors from both teams always introduced themselves as icddr,b employees to the study participants. The census data collectors, who visited each of the study households only once every 6 months, mentioned that before each census round as the area where they would conduct the survey would change for each of the data collectors. According to the field supervisor, this was practiced to avoid repeated mistakes (if there were any) made by the same data collector in the same area throughout the study period. It was unlikely that the same census data collector visited the same household or the compound twice in a year.

By contrast, for the convenience of some of the monthly data collectors, some of the areas for data collection were fixed. Although they visited a household only once during the whole study period, sometimes they had to go back to the same compound to interview a different household several times. As one of the data collectors mentioned, “We never visited the same household twice throughout the study period but sometimes we had to visit different households within the same compound several times. Depending on random selection of households sometimes we had to visit the same compound twice in a week for interviewing different households.”

Data collectors from both teams asked study participants about diarrhea within the last 48 hours (census) or 2 days (monthly surveys) in a similar way. Both teams explained to the study participants how they should count the 48 hours or 2-day period from the time of interview and mentioned that ≥ 3 loose stools within 24 hours would be considered as diarrhea.

According to both census and monthly survey data collectors, the study participants were aware that the intervention products were distributed in the community by icddr,b through the DSK. Several data collectors from the census team mentioned that study participants from the control or vaccine-only group sometimes asked them why they were not given the behavior change intervention products. Study participants from the vaccine-plus-behavior-change group sometimes would request that census data collectors convey messages to the DSK personnel about product-related problems (breakage/leakage) or requirements (running out of liquid chlorine). In response, the data collectors would tell them to directly talk to the DSK personnel, but that if they came across any DSK personnel during data collection, they would convey the message. The monthly survey team similarly received both complaints and compliments about behavior change intervention products. Study participants expected monthly data collectors to fix hardware-related problems, or convey messages to DSK personnel to come and fix the problem. The monthly data collectors conveyed these messages to two of the icddr,b field staff who worked directly with the DSK managing hardware-related problems in the field.

## DISCUSSION

In this study, we observed an impact of the behavior change intervention on reported diarrhea for children aged ≤ 5 years in the monthly survey but not in the census group. Similar to diarrhea prevalence data collected through census surveys, there was no impact of the intervention on objectively assessed diarrhea-associated hospitalization. This may suggest that the reported diarrhea prevalence data collected through the census may be more reliable than the data collected through the monthly surveys. However, this interpretation assumes that there is correlation between diarrhea hospitalizations and less severe, community-based self-reported diarrhea. This assumption may or may not be correct, given the seasonal patterns of different pathogens that may produce diarrhea of different severity at the community level.^39^

The reasons for observing the impact of the intervention in the monthly surveys are unclear but could be due to bias rather than an actual intervention effect. The presence of observer bias in non-blinded studies has been frequently reported. Hróbjartsson and others^31^ conducted a systematic review of randomized clinical trials with both blinded and non-blinded assessment of same subjective measurement scale outcomes with an aim to assess the presence of observer bias and reported that the non-blinded assessors exaggerated the pooled effect size by 68%. In our study, the monthly survey team was directly supervised by researchers involved in developing and implementing the behavioral interventions, and the focus of this team was assessing the uptake of behavioral interventions. Given the non-blinded nature of this study, these assessors may have been predisposed to expect lower diarrhea prevalence in the intervention group, and consciously or unconsciously may not have recorded information on diarrhea.^40^ By contrast, the census data collectors may have been comparatively more neutral in collecting diarrhea data considering the vaccine implementation team of researchers supervised them and their focus of data collection was updating household demographic information rather than assessing the uptake of behavior change interventions. However, group discussions with the monthly survey data collectors did not reveal any information on perceived pressure to indicate the presence of observer bias; so if this bias was operating, it may have been unconscious.

Other possible explanations for the difference in the census and monthly survey data include minor differences in methodology, framing of the questions to collect information on diarrhea, and sampling variability. For the monthly surveys, data collectors did not visit households more than once within the study period, but may have visited the same compound several times even within a week. As our interventions were mostly implemented at the compound level, it is possible that repeated visits to the same compound within a short time interval combined with the considerable amount of time spent assessing behavioral intervention uptake may have alerted some participants to the fact that reduced diarrhea was a “desirable outcome” of the intervention. This could have influenced reporting of diarrhea because of social desirability bias,^41,42^ Hawthorne effect,^43,44^ or courtesy bias.^20^

In census and monthly surveys, a similar recall period and diarrhea case definition were used, although the framing of the recall period was slightly different (diarrhea within the last 2 days in monthly surveys and within 48 hours in the census). However, it is unlikely that this created any difference in diarrhea prevalence measurement because both data collector teams similarly explained how they counted “2 days” or “48 hours” period at the time of interview. In both surveys, we specified diarrhea as being defined as ≥ 3 loose stools within 24 hours, which is similar to what has been suggested by the WHO^45^ and has been adopted in many other studies.^46–49^ As two different teams collected data from different study participants at different time points, sampling variability could be another possible reason for differences in the intervention impact on reported diarrhea.

Collecting information on reported diarrhea is an easy and inexpensive way of assessing the impact of behavioral interventions, but this presumes that such data are sufficiently valid to support inference. Our study findings add further evidence of the subjectivity of self-reported diarrhea in non-blinded trials that can affect assessment of the intervention impact.^33^ Keeping the data collection interview period brief and avoiding assessing health outcome and intervention uptake at the same time could minimize the risk of bias. These study findings highlight the importance of measuring objective outcomes when assessing non-blinded trials and comparing these with subjective outcome measures.

## Supplementary Files

Supplemental tables
