# Supplementary material for: Inconsistency in Diarrhea Measurements when Assessing Intervention Impact in a Non-Blinded Cluster-Randomized Controlled Trial
Source: Am J Trop Med Hyg. 2019 Jun 3;101(1):51–8. doi: 10.4269/ajtmh.18-0872 (PMC6609177; doi:10.4269/ajtmh.18-0872)
Supplement: Supplementary file 1 [file tpmd180872.SD1.pdf]

Supplementary table 1: Diarrhea-associated hospitalization rates and hazard ratios during study period by intervention groups among children ≤5years*

Group	No. of people	No. of person years (1000)	No. of hospitalization	Hospitalization rate (individual group)/ 1000 person-years (95% CI)	Hazard ratio (95% CI)	P-value
Control	20,034	22.7	847	39.4 (33.1, 47.2)	1.0	-
Vaccine-only	21,846	25.0	928	39.3 (34.5, 44.8)	1.0 (0.81, 1.23)	0.98
Vaccine-plus-behavior-change group	20,817	23.6	969	43.3 (36.7, 51.5)	1.1 (0.86, 1.40)	0.44

*Results adjusted for cluster randomized design

Supplementary table 2: Age stratified distribution of participants in the census survey*

Age	Vaccine-only N=142,879 %	Vaccine plus behavior change N=140,202 %	Control N=137,451 %
≤1 year	7.0	7.2	7.2
>1 to ≤2 years	1.9	1.9	1.9
>2 to ≤3 years	2.0	1.9	2.0
>3 to ≤4 years	1.9	1.9	1.9
>4 to ≤5 years	1.9	1.8	1.8
>5 years	85.3	85.3	85.2

* Some categories do not sum to 100% because of rounding

Supplementary table 3: Age stratified distribution of participants in the monthly-survey*

Age	Vaccine-only N=13,914 %	Vaccine plus behavior change N=27,059 %	Control N=14,236 %
≤1 year	3.5	3.7	3.3
>1 to ≤2 years	2.0	1.6	2.1
>2 to ≤3 years	1.9	1.8	1.9
>3 to ≤4 years	2.2	1.9	2.1
>4 to ≤5 years	1.7	1.6	1.7
>5 years	88.6	89.4	89.0

* Some categories do not sum to 100% because of rounding
